# Short-term effect of kinesiology taping on pain, functional disability and lumbar proprioception in individuals with nonspecific chronic low back pain: a double-blinded, randomized trial

**DOI:** 10.1186/s12998-020-00349-y

**Published:** 2020-11-20

**Authors:** Soheila Abbasi, Mohammad-Reza Hadian Rasanani, Nastaran Ghotbi, Gholam Reza Olyaei, Ali Bozorgmehr, Omid Rasouli

**Affiliations:** 1 Department of Physiotherapy, School of Rehabilitation, Tehran University of Medical Sciences, (TUMS), Tehran, Iran; 2 Postgraduate Department, School of Rehabilitation, Tehran University of Medical Sciences, International, Brain and Spinal Injury Research Center (BASIR), Institute of Neuroscirnce, P.O. Box: 111551683, Tehran, Iran; 3 Rehabilitation Research Center, Department of Physical Therapy, School of Rehabilitation Sciences, Iran University of Medical Sciences, Tehran, Iran; 4 Department of Public Health and Nursing, Faculty of Medicine and Health Sciences, Norwegian University of Science and Technology (NTNU), Trondheim, Norway

**Keywords:** Pain level, disability, Proprioception, Low Back pain, Kinesiotape, Sham

## Abstract

**Background:**

This study aimed to evaluate the effect of kinesiology taping (KT) on lumbar proprioception, pain, and functional disability in individuals with nonspecific chronic low back pain (CLBP).

**Methods:**

Thirty individuals with nonspecific CLBP participated in this double-blinded, randomized clinical trial from July 2017 to September 2018. The participants were randomized into two groups: KT (*n* = 15) and placebo group (n = 15). KT was applied with 15–25% tension for 72 h, and placebo taping was used without tension. Lumbar repositioning error was measured by a bubble inclinometer at three different angles (45° and 60° flexion, and 15° extension) in upright standing. Pain and disability were assessed by the Short-Form McGill Pain Questionnaire and Oswestry Disability Index, respectively. All measurements were recorded at baseline and 3 days after taping.

**Results:**

Pain and disability scores reduced 3 days after taping in the KT group with large effect sizes (*p* < 0.05). Only the total score of pain was significantly different between the groups 3 days after taping and improved more in the KT group with a large effect size (*p* < 0.05). However, lumbar repositioning errors were similar between the groups after 3 days (*p* > 0.05). Also, only constant error of 15° extension showed a moderate correlation with disability (r = 0.39, *p* = 0.02).

**Conclusion:**

KT can decrease pain and disability scores after 3 days of application. Although placebo taping can reduce pain, the effect of KT is higher than placebo taping. The findings do not support the therapeutic effect of KT and placebo taping as a tool to enhance lumbar proprioception in patients with nonspecific CLBP.

**Trial registration:**

The study prospectively registered on 21.05.2018 at the Iranian Registry of Clinical Trials: IRCT20090301001722N20.

## Background

It is known that proprioception is necessary for the control of human movement [1]. Proprioception is described as the joint’s sensation, position, and movement; also, the sense of force, effort, weight, and perceived timing associated with muscular contraction [2]. Proprioceptive inputs are derived from afferent information received from muscle, joint, and skin receptors [2]. These receptors have different roles depending on the range at a given joint; for example, previous studies have found that joint receptors to be activated near the end of the joint range, while muscle spindles provide afferent inputs throughout the physiologic ranges [3].

It is challenging to measure proprioception because of its complex function. In previous studies, proprioception has been measured by force–plate analysis [4], electromyographic (EMG) activity [5], and position sense [6]. Position sense is defined as the ability to perceive the movement or orientation of a body segment in space. Some studies have used repositioning error (RE) or position sense to measure proprioception in joints [6, 7]. There is evidence that individuals with chronic low back pain (CLBP) have reduced proprioceptive ability and larger lumbar RE in the lumbar region [7, 8], but some studies have found no significant difference between subjects with CLBP with pain-free participants [9, 10]. If proprioceptive deficits exist, rehabilitation programs should be designed to improve proprioception; however, there has been little research to support this.

In recent years, the use of a therapeutic tool called kinesiology tape (KT) has increasingly become popular to use for musculoskeletal disorders. KT is made up of colorful elastic cotton strips with an acrylic adhesive that may be stretched to up to 140% of their original length [11]. KT is assumed to have several benefits [12], including (1) pain reduction through neurological suppression, (2) reposition of subluxated joints by decreasing abnormal muscle tone, (3) to create more space by lifting fascia and soft tissue to improve circulation, (4) correcting muscle function by strengthening weak muscles (5) and providing sensory stimulation to improve proprioception [12, 13].

The compressive and stretching effect of KT provides additional cutaneous stimulations, and these stimulations transfer more information regarding the joint position and movement to the central nervous system (CNS) for integration resulting in increased proprioception [2, 14]. However, there have been controversial results regarding the effect of KT on proprioception.

Some studies have shown improved proprioception through augmented cutaneous sensory stimulations provided by KT [14, 15], whereas others have found no changes in proprioception using KT [13, 16]. Therefore, CLBP individuals with poor proprioception might benefit from the application of KT.

As yet, there is a lack of research exploring the effect of KT on lumbar proprioception. The purpose of the present study was to explore the short-term effect of KT on lumbar proprioception, pain, and functional disability in individuals with nonspecific CLBP. The associations between pain intensity, disability, and lumbar RE were also evaluated in these individuals**.** It was hypothesized that applying KT on the lumbar spine would improve lumbar proprioception, pain, and disability compared to the placebo group in individuals with nonspecific CLBP.

## Methods

### Study design

This study was a parallel double-blinded (assessor and participants), prospective randomized trial. The study was conducted at the Tehran University of Medical Sciences in accordance with the principles of the Declaration of Helsinki. The Ethical Committee at the Tehran University of Medical Sciences has approved this study, and it was prospectively registered at the Iranian Registry of Clinical Trials: IRCT20090301001722N20 at the website https://www.irct.ir/. All participants signed a consent form before entering the study.

### Participants

**Fig. 1:**
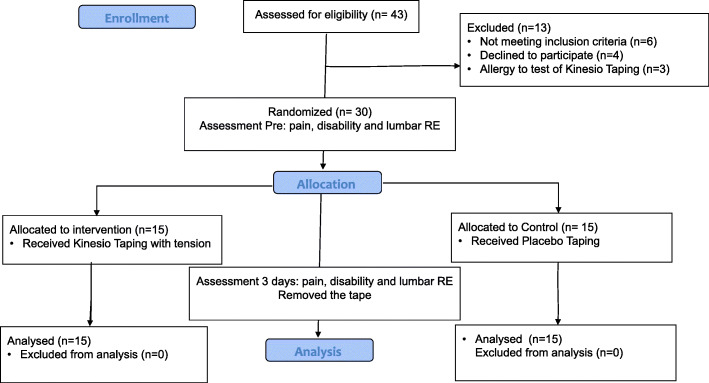
The flow of participants recruitment through the study

A total of 43 individuals were screened for this study. Thirteen were excluded for the reasons presented in Fig. 1. Thus, 30 individuals with nonspecific CLBP (15 males, 15 females), ages 25–50 years, participated in the study. All participants were referred from two public physical therapy clinics associated with the Tehran University of Medical Sciences from July 2017 to September 2018. Based on the definition of nonspecific CLBP, the inclusion criteria were localized back pain between the 12th rib and the gluteal folds lasting more than 3 months. The individuals were excluded if they had any of the following criteria: the history of pain radiating further than the buttock, sciatica or other radicular involvement, spinal surgery, nerve root compression, neurological deficits, rheumatic diseases, diabetes, mental disorders, pregnancy, lower extremity injuries, or neuromuscular diseases. Participants were also excluded if they presented signs of allergy to KT during a test conducted before the initial evaluation. KT without tension was used to test if volunteers had any allergic reactions to taping, and three individuals (of 43 individuals) had allergic reactions, e.g. redness and itching, to KT; therefore, they were excluded from the study. No one showed any allergic reactions before or after the removal of the tape among those 30 participants who completed the testing.

### Procedure

Individuals were randomly assigned to either placebo group (KT without tension) or experimental group (KT with tension) according to a randomization scheme generated by a computer. Individuals with odd numbers were allocated to the placebo group, and individuals with even numbers were allocated to the KT group. The allocation of the participants was concealed by using sequentially numbered, sealed, and opaque envelopes. Blinded investigator (investigator 1) conducted the data collection and analysis, and investigator 2 conducted enrolling participants and intervention to minimize potential sources of bias. Also, all participants were unaware of their group. Figure 1 displays the recruitment process for this study. All outcomes were collected in a biomechanics laboratory at the Tehran University of Medical Science.

### Intervention and placebo group

**Fig. 2:**
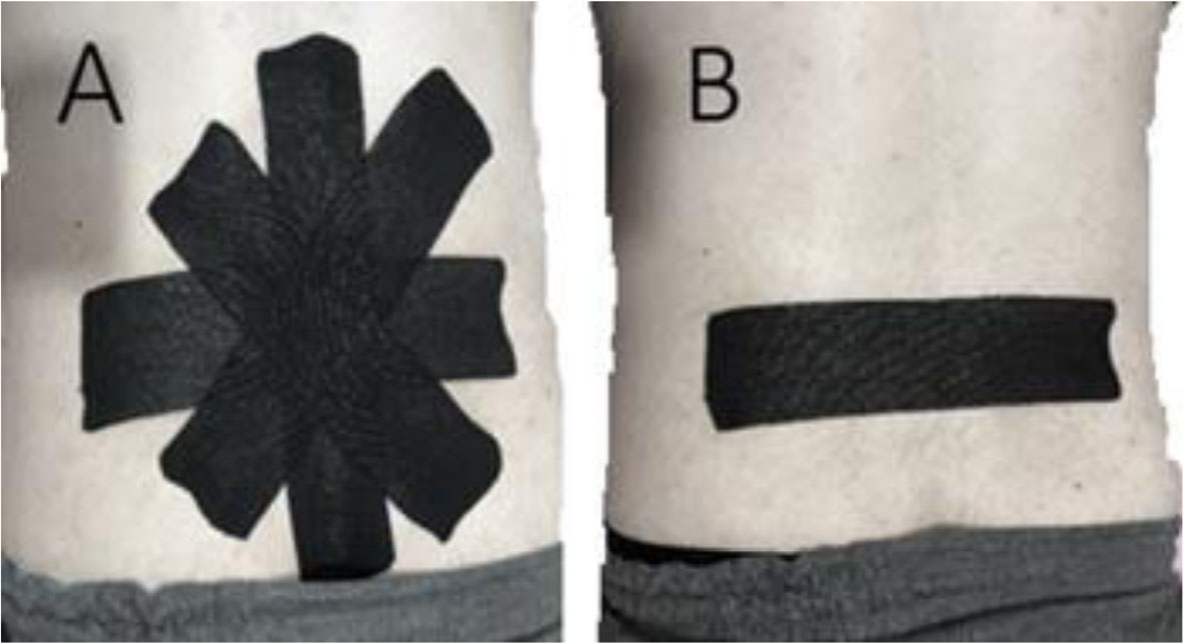
Star shape Kinesio tape application that four I-strips of KT was placed over the point of maximum pain in the lumbar area (**a**), placebo taping that single I-strip applied transversely immediately above the point of maximum lumbar pain (**b**)

In this study, waterproof, adhesive tape (NST-05002, made in Korea) with a width of 5 cm and a thickness of 0.5 mm was used. The experimental group received a standardized KT application in sitting position for 72 h. We had chosen applying this method of KT (star shape) in sitting position following the method used by previous similar studies [17, 18]. Four I-strips, including one vertical, one horizontal, and two at 45° angles to vertical strip, were attached with 15–25% tension overlapping in a star shape over the point of maximum pain in the lumbar area. The central part of the strips was applied before the ends by pressing and adhering, and all strips have crossed at the central point of the tape (Fig. 2a). It seems that the star shape of KT with 15–25% tension in the lumbar region is more effective than other methods in reducing pain and disability and stimulation of mechanoreceptor in subjects LBP [12, 18, 19].

The placebo group received a single I-strips of the same tape without tension transversely on the maximum pain point of the lumbar region (placebo taping) (Fig. 2b). After 3 days, the taping was removed for both groups, and re-evaluation was performed by investigator 1.

### Outcomes measures

Assessments were taken at baseline and 3 days after the intervention. All participants completed measures of pain intensity by the Short-Form McGill Pain Questionnaire (SF-MPQ) [20], functional disability by Oswestry Disability Index (ODI) [21], and lumbar RE using bubble inclinometer before and after the intervention.

The SF-MPQ consists of three parts, including 15 descriptors of pain (11 from sensory categories, and 4 from affective categories), Visual Analogue Scale (VAS), and present pain intensity (PPI). Descriptors are rated based on pain severity on a four-point scale (0 = none, 1 = mild, 2 = moderate, 3 = severe). The scores of sensory and affective (S/A) part are calculated by summing sensory and affective item values, as the total score. The second part is VAS, which is a 10-cm horizontal line ranging from “no pain” to “worst possible pain.” Patients present the severity of their pain by marking the line. After that, pain intensity was calculated from zero to the marked point by the patient in centimeters. The third part of the SF-MPQ is the PPI, which is a six-point verbal rating scale ranging from none (0) to the worst excruciating (5). The patients choose the word that best describes the overall intensity of their pain at the present time; then, the questionnaire is completed. The MPQ as a multidimensional tool is designed to assess sensory, affective, and evaluative dimensions of pain [20].

Considering the effects of KT on various aspect of pain (i.e., sensory, affective and evaluative dimensions of pain) [22], it seems that the MPQ could better show the effects of KT on pain after taping than other tools such as VAS alone or Numerical Rating Scale (NRS). Also, the conflicting results about the effects of KT on pain in individuals with LBP probably resulted from it. The current study was the first study that investigated the effects of KT on pain using SF-MPQ as a multidimensional tool in subjects with nonspecific CLBP.

**Fig. 3:**
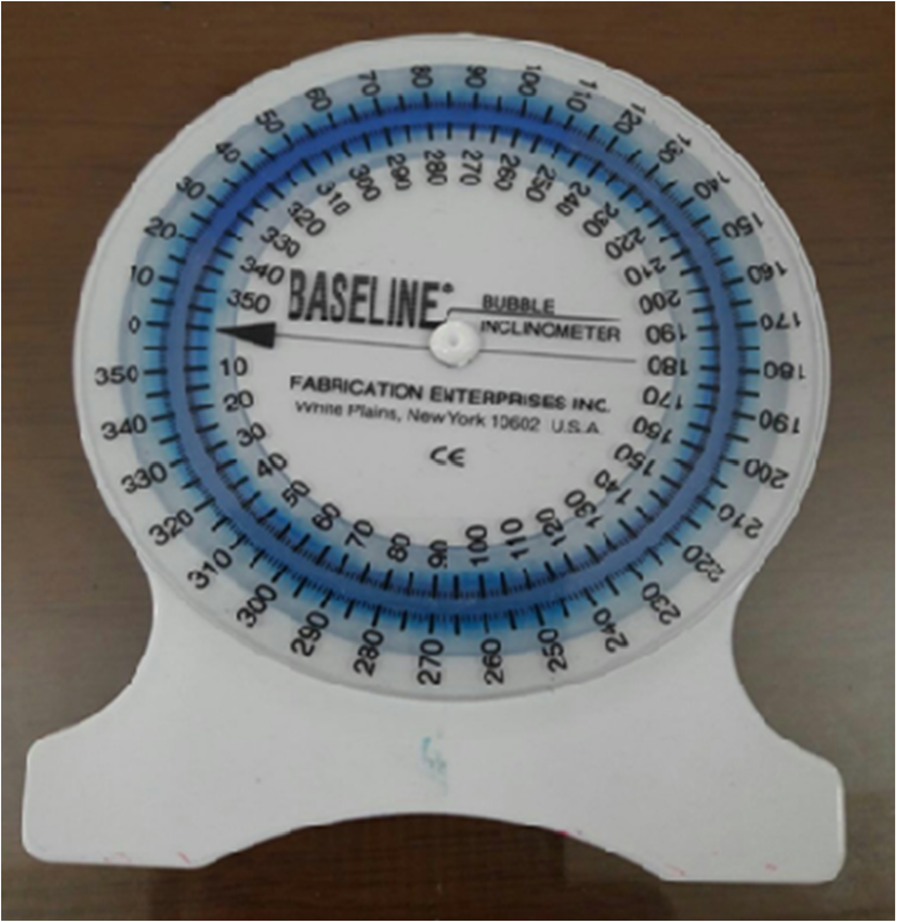
The bubble inclinometer

In addition, lumbar RE, as an indirect measure of proprioception, was determined using a bubble inclinometer because of its reasonable price, the facility of application, accessibility, and non-invasiveness. Results of previous studies showed that measuring the lumbar range of motion (ROM with a bubble inclinometer was valid and reliable [23–25]. The bubble inclinometer is circular, fluid-filled disc devices, with an adjustable scale to permit zeroing (Model 10,602 built by Fabrication Enterprise Inc. USA) (Fig. 3).

The participant wore only shorts to reduce sensory cues from clothing. First, the therapist marked the T12-L1 and S1 spinal levels by a marker. Two bubble inclinometers were used simultaneously for measuring the flexion and extension of lumbar ROM in standing position. The participant stood with feet 15 cm apart and arms at their sides while looking forward. One inclinometer was placed at T12-L1, and the other one located over the sacrum. The inclinometers were set as close to 0 degrees as possible. While holding the inclinometers, the participant was asked to bend forward and keep the knees straight. Maximum values in both inclinometers were recorded. The actual range of Lumbar flexion was calculated by subtracting the records from S1 from the device placed over T12-L1 [23].

The same landmarks and procedures that were defined for the flexion technique were used for measuring lumbar extension. Both inclinometers were located over the skin marks while holding the inclinometers, and the therapist instructed the participant to bend backward. The angles were recorded on the inclinometers and subtracted [23].

Repositioning accuracy was assessed with participants trying to reproduce the target position.

Three different target position was chosen. Participants were asked to reproduce two different trunk positions from the neutral spinal posture to 45° and 60° flexion and one position from a neutral spinal posture to 15° extension. These angles were chosen because previous research indicated that participants with LBP have greater difficulties in reproducing 45° and 60° lumbar flexion and 15° lumbar extension than healthy ones [8, 26].

Each participant was positioned into an upright neutral starting position. This position was such that the anterior superior iliac spine and the posterior superior iliac spine were aligned in the horizontal plane using a pelvic inclinometer. To determine the maximum available lumbar ROM and whether the participant was able to perform the experimental tasks, each participant was instructed to move into flexion as much as possible, then the participant moved into extension as much as possible. Each participant was positioned in 45° of lumbar flexion for 10 s and was asked to remember the position because he or she would be asked to reproduce this position with closed eyes. Next, the participant returned to the neutral position and then was given the verbal instruction of reproducing the target position as accurately as he or she could. The participant reported to the therapist on reaching the target position as perceived by him or her. The participant was required to hold the final position for 3 s; then, the reproduced position was recorded by the therapist. The same procedures were defined for 60° lumbar flexion and 15° lumbar extension. The tests were randomly performed, and each test was repeated three times with a 30 s rest between each trial; then, the average of them was calculated for the analysis. The participants were allowed to practice the test protocol once before the test. No feedback was given during testing.

At baseline, all outcome measures were assessed for each participant before the intervention. Then, the participants were taped, and re-evaluation was performed after 3 days. RE was defined as the constant error (CE) between the target position and the actual measured angles that CE indicates error towards a particular direction (positive or negative and the absolute error (AE) was the unsigned deviation from the target position, thus reflecting absolute repositioning error, irrespective of error direction. Both errors were assessed before and after the intervention.

### Statistical analysis

The sample size calculation was performed using G*Power version 3.1.9.2 [27]. This was determined using a power of 0.8, an alpha value of 0.5. It resulted in a required sample size of 13 subjects in each group. Assuming an attrition rate of 10% due to dropouts and technical problems, it was determined that we would require 15 participants in each group.

Statistical analyses were performed using IBM SPSS Statistics for Windows, version 22.0 (IBM Corp., Armonk, NY, USA). The normality of data was assessed, and all variables followed a normal distribution. In this study, all variables (pain, functional disability, CE, and AE values) were presented as mean (standard deviation). All variables were compared between the groups using independent sample t-tests. Paired t-tests were also applied to find the differences between before and 3 days after the taping in each group. Cohen d was reported as the estimates of effect size, with the classification of *small* (*d* =  0.2), *medium* (*d* =  0.5), and *large* (*d* ≥ 0.8) [28]. The correlations between pain intensity, disability, and RE were assessed using Pearson correlation coefficient before taping for all participants. The correlations were rated as strong (> 0.7), moderate (> 0.5), or weak (< 0.3) [29]. The level of significance was set at *p* < 0.05.

## Results

The demographic characteristics of the participants are shown in Table 1. The participants in the placebo and KT groups had a mean age of 42 and 44 years, respectively, with mild to moderate pain and disability. Half of the participants were females. After randomization, all participants were assessed, and none of the participants were excluded from the study. The groups were comparable at baseline, and there were no significant differences between the two groups regarding age, gender, BMI, pain **(**Total score of pain (S/A), VAS, PPI), or disability (ODI) (Table 1).

**Table 1:** Characteristics of participants in each group

Variable	Kinesio taping (***n*** = 15)	Placebo (n = 15)	***P***-value
**Age (years)**	44.3 ± 3.6	42.1 ± 6.9	0.274^†^
**Gender (female)**	9 (60%)	6 (40%)	0.439^‡^
**BMI (kg/m2)**	24.9 ± 3.5	24.8 ± 3.6	0.890^†^
**Total score of pain (S/A)**	18.2 ± 6.9	15.4 ± 5.4	0.296^†^
**Pain (VAS)**	5.6 ± 1.4	4.7 ± 1.1	0.062^†^
**PPI**	2.2 ± 0.7	2.1 ± 0.4	0.549^‡^
**Disability (ODI)**	21.1 ± 6.1	19.2 ± 6.1	0.397^†^

*BMI* Body Mass Index, *VAS* Visual Analogue Scale, *ODI* Oswestry Disability Index, on a percentage scale, score from 0 to 20% indicate a minimal disability, 21–40% indicate a moderate disability, 41–60% severe disability, 61–80% for crippled and 81–100%. *S/A* Sensory and Affective, *PPI* Present Pain Intensity, *AE* Absolute Error, *CE* Constant Error. Data are presented as means and standard deviation (Mean ± SD). † Independent sample t-test, ‡ Chi-square test

### Effect of intervention

There were no significant differences in pain (total score of pain (S/A), VAS and PPI), functional disability or RE (AE and CE) at the angles (45° and 60° flexion and 15° extension) between the groups at baseline (*P* > 0.05). After 3 days of taping, the pain (total score of pain (S/A), VAS, and PPI) and functional disability scores significantly reduced in the KT group with large effect sizes (d > 0.8), also PPI significantly decreased in the placebo group with a large effect size (*P* < 0.05, d = 0.8). However, the difference between the groups was significant only for the total score of pain (S/A) 3 days after taping and improved significantly more in the KT group than the placebo group with large effect size (*P* < 0.05) (Table 2).

**Table 2:** Mean (SD) for All outcomes for each group at baseline and 3 days after taping and as mean (95% confidence interval) for within and between-group differences

Outcome	Groups with CLBP Mean (SD)					Difference within groups (95% CI)			Difference between groups (95% CI)		Effect size (Cohen’s d)^±^
	Baseline		3 days after taping		3 days after taping minus Baseline	Effect size (Cohen’s d)^±^	3 days after taping minus Baseline	Effect size (Cohen’s d)^±^	Baseline	3 days after taping	3 days after taping
	KT n = 15	Placebo n = 15	KT n = 15	Placebo n = 15	KT n = 15		Placebo n = 15		KT minus Placebo	KT minus Placebo	KT minus Placebo
Total score of pain (S/A)	18.2 (6.9)	15.4 (5.4)	10.6 (5.47)	15.73 (5.32)	−7.6^*^ (4.14, 11.05)	0.12	0.27 (− 0.83, 0.3)	0.36	2.73 (−1.94, − 7.4)	− 5.13^**≠**^ (− 9.17, 1.09)	0.95
Pain (VAS)	5.6 (1.4)	4.7 (1.1)	4.33 (1.39)	3.8 (1.32)	−1.33^*^ (0.83, 1.83)	0.96	−0.93 (− 0.03, 1.9)	0.22	0.93 (− 0.04, − 1.91)	0.53 (− 0.48, − 1.55)	0.39
PPI	2.2 (0.7)	2.1 (0.4)	1.33 (0.72)	1.53 (0.51)	−0.87* (0.45, 1.27)	0.86	−0.53* (0.24, 0.81)	0.8	0.13 (−0.34, − 0.6)	−0.2 (− 0.67, − 0.27)	0.32
Disability (ODI)	21.1 (6.1)	19.2 (6.1)	18.8 (4.7)	19.2 (6.39)	−2.26* (0.76, 3.76)	0.85	0.0 (−1.1, 1.1)	0.01	1.86 (− 2.57, −6.31)	−0.4 (−4.58, −3.78)	0.07
AE- 45° flexion	3.7 (2.73)	2.4 (2.33)	2.46 (2.48)	3.33 (4.04)	−1.24 (− 1.0, 3.46)	0.35	0.93 (−2.67, 0.8)	0.16	1.3 (−0.6, 3.2)	−0.86 (−3.37, 1.64)	0.25
AE-60° flexion	4.46 (3.31)	4.46 (3.88)	2.76 (2.7)	3.76 (2.81)	−1.7 (−0.86, 4.26)	0.21	−0.7 (− 0.83, 2.23)	0.34	0.0 (−2.7, 2.7)	− 1.0 (−3.06, 1.06)	0.36
AE-15° extension	3.26 (3.88)	2.56 (1.78)	3.36 (3.19)	2.66 (2.24)	0.1 (−3.01, 2.81)	0.02	0.1 (−1.46, 1.26)	0.08	0.7 (−1.56, 2.96)	0.7 (−1.32, 2.72)	0.25
CE-45° flexion	−0.23 (4.7)	1.60 (2.97)	−0.8 (3.46)	2.26 (4.76)	1.03 (−3.51, 1.45)	0.23	0.66 (−2.67, 1.34)	0.21	−1.83 (−4.77, 1.1)	− 1.46 (− 4.58, 1.65)	0.73
CE-60° flexion	−0.53 (6.55)	2.46 (5.47)	0.63 (3.88)	2.23 (4.21)	1.16 (−4.58, 2.24)	0.03	−0.23 (−1.52, 1.99)	0.07	−3.0 (−7.16, 1.16)	−1.60 (− 4.63, 1.43)	0.39
CE-15° extension	−2.86 (4.21)	0.83 (3.08)	−1.1 (4.52)	−0.06 (3.55)	1.76 (−5.25, 1.72)	0.33	−0.89 (− 0.71, 2.51)	0.45	−3.70 (6.45, − 0.94)	−1.03 (− 4.07, 2.0)	0.25

*CLBP* Chronic Low Back Pain, *95% CI* 95% Confidence Interval, *KT* Kinesio Taping group, *ODI* Oswestry Disability Index, *VAS* Visual Analog Scale, *S/A* Sensory and Affective, *PPI* Present Pain Intensity, *AE* Absolute Error, *CE* Constant Error. Values are expressed as mean (SD) for baseline and 3 days after taping and as mean (95% confidence interval) for within- and between-group differences

^*^Significant differences (*P* < 0.05) within group

^**≠**^Significant differences (*P* < 0.05) between group

±Cohen classified effect sizes as *small* (*d* =  0.2), *medium* (*d* =  0.5), and *large* (*d* ≥ 0.8)

No significant between-group or within-group differences were observed for the RE (AE and CE) in any of the angles (45° and 60° flexion and 15° extension) at 3 days (*P* > 0.05) (Table 2).

### Correlations with RE

According to the findings, only CE of 15° extension showed a significant moderate positive correlation with disability (r = 0.39, *P* = 0.02) (Table 3).

**Table 3:** The correlations between repositioning error values (constant error and absolute error), pain, and disability

	Pain (Total score of pain) (S/A)	Pain (VAS)	Pain (PPI)	Disability (ODI)
CE				
45° flexion	r = 0.14	r = −0.03	r = −0.12	r = − 0.15
	*p* = 0.435	*p* = 0.853	*p* = 0.501	*p* = 0.427
60° flexion	r = −0.04	r = − 0.17	r = − 0.12	r = − 0.11
	*p* = 0.815	*p* = 0.359	*p* = 0.513	*p* = 0.560
15° extension	r = 0.19	r = −0.32	r = −0.06	r = 0.39
	*p* = 0.293	*p* = 0.080	*p* = 0.721	*p* = 0.029^*^
AE				
45° flexion	r = −0.23	r = 0.12	r = −0.30	r = − 0.16
	*p* = 0.216	*p* = 0.50	*p* = 0.106	*p* = 0.377
60° flexion	r = −0.33	r = 0.22	r = 0.20	r = 0.17
	*p* = 0.074	*p* = 0.234	*p* = 0.282	*p* = 0.366
15° extension	r = −0.10	r = − 0.02	r = − 0.17	r = − 0.23
	*p* = 0.601	*p* = 0.912	p = 0.366	*p* = 0.209

*ODI* Oswestry Disability Index, *VAS* Visual Analog Scale, *S/A* Sensory and Affective, *PPI* Present Pain Intensity, *AE* Absolute Error, *CE* Constant Error

* Statistically significant: (*P*-value < 0.05)

## Discussion

The current study aimed to examine the effect of KT application on lumbar proprioception, pain, and functional disability in individuals with nonspecific CLBP. The results showed that pain (Total score (S/A), VAS and PPI) and disability scores significantly improved after 3 days of taping in the KT group with large effect sizes, but no significant differences in RE (AE or CE) were found in any of the three angles (45° and 60° flexion and 15° extension). In the placebo group, only PPI was significantly reduced after 3 days of placebo taping without tension. Also, only the total score of pain (S/A) showed significant differences between the groups with a large effect size.

Participants in the KT group had mild to moderate pain levels and showed approximately 23% reduction of pain over 3 days; therefore, possibly over a longer period, a greater reduction would have been achieved. However, KT is typically worn for 3–5 days.

There are several studies investigated the effect of KT on pain and disability in individuals with LBP [17, 30, 31]; however, there is a large variability in a combination of KT with other therapies, such as exercise program, manual therapy, and traditional physical therapy. Some studies compared the effect of KT to placebo taping on pain and disability. In line with our findings, some studies showed that the levels of pain and disability significantly decreased after taping [18, 32–34], and some reported that KT is better than placebo taping in patients with CLBP [32, 34, 35]. On the other hand, some studies found no significant decrease in pain or disability after the intervention [36, 37], and that the application of KT was not better than placebo taping for patients with CLBP [31, 33, 37], which is contradicted with our findings. In the current study, similar to previous studies [18, 32, 33, 35, 36], the placebo taping had therapeutic effectiveness beyond the placebo. It seems that applying placebo taping without tension even with one strip can create some physiological.In the current study, KT only reduced the total score of pain (S/A) in the KT group compared to the placebo group. It indicates that KT can affect other aspects of pain (i.e., sensory, affective) that placebo taping could not affect. Because of the PPI reduction in the placebo group, it seems that the application of placebo taping could reduce pain intensity due to a placebo effect in the patients with CLBP. Based on our results, both KT and placebo taping can reduce pain in patients with CLBP, but the effects of KT are higher than placebo taping.

Some physiological mechanisms of KT effects have been proposed. Pain reduction after applying KT with tension may be due to lifting the skin and enhancing subcutaneous space and, as a result, reduced activation of pain receptors, also possibly activates descending inhibitory system. Also, in the gate control theory of pain, tactile stimulation of KT would reduce the afferent signal of large-diameter non-nociceptive fibers resulting in a reduction of pain [18, 38, 39]. In addition, some positive effects of KT may indirectly affect pain and disability improvement, such as normalize muscle tone, improvements of postural control, range of motion, circulation, and proprioception [40–43].

The previous studies never evaluated the effects of KT on pain using SF-MPQ as a multidimensional tool in individuals with nonspecific CLBP, and they measured pain by VAS [18, 32, 34] or NRS [33, 35–37]. The current study is the first to assess the effects of KT on pain using SF-MPQ in individuals with nonspecific CLBP.

Also, there were no significant differences (between-group or within-group) for lumbar RE (AE and CE) in the angles (45° and 60° flexion and 15° extension). To the best of our knowledge, this is the first study to evaluate the effect of KT on lumbar proprioception in patients with LBP. Several studies have evaluated the effect of KT on proprioception in peripheral joints, especially knee and ankle in injured or non-injured individuals [11, 13–15] that are conflicting with our results. Several explanations may explain our results. Based on our results, it seems that placebo taping may affect and reduce pain, albeit slightly. Therefore, the lack of non-taping group and the effect of placebo taping may have affected the results of between-group comparison in this study.

A systematic review reported that patients with LBP have impaired lumbar proprioception compared with controls when measured actively in sitting positions [4]. In our study, lumbar proprioception was actively measured in standing positions that may have affected the results. Also, various methods of measuring lumbar proprioception exist, including joint RE, the threshold to detection of passive motion, and directional motion perception [4]. In the current study, lumbar proprioception was measured by repositioning error. However, the best method of measuring proprioception is still unclear. In addition, in the previous studies, target positions for repositioning ranged from neutral lumbar spinal posture to target angles in pelvic tilting and lumbar flexion, extension, lateral flexion, and rotation in patients with LBP [4, 6, 7]. This study reproduced two different trunk positions from neutral to 45° and 60° flexion and one position from neutral to 15°extension. Perhaps within other ranges, individuals show more proprioception deficits and/or improvements after KT.

The potential mechanism by which KT improves proprioception is not yet understood. Some authors have hypothesized that cutaneous feedback supplied by KT could be increased. Applied pressure and stretching due to KT application on the skin at extremes of motion, similar to joint mechanoreceptors, can also stimulate cutaneous mechanoreceptors and signal information of joint movement or joint position [13, 14, 44]. Konishi et al. (2013) confirmed that KT could counter quadriceps femoris weakness due to attenuated la afferent activity [44].

It seems that tactile stimulation of KT was not enough for improvements of proprioception may be due to short-term assessment or method of taping in our study. There are direct relationships between impaired proprioception, pain, and reduced quality of life [45]. In the current study, pain and disability improved in the patients; therefore, there would be improvements in proprioception as well as pain and disability reduction in the KT group.

It has been proposed that proprioceptive deficits may lead to trunk muscle dysfunction also may cause alterations in normal afferent inputs from the affected muscles. In neutral posture, muscle afferents could be considered as primary contributors to position sense because ligaments are under minimal tension [3, 5]. In contrast, previous studies reported that KT could normalize lumbar muscle function and postural control in patients with LBP [17, 41, 43], and it is thus expected to improve lumbar proprioception.

The tension of KT is described as one of the critical factors for successful implementation. Theoretically, both 75 and 100% of tension are used to support weak muscle or correction of joint position, 25–50% for muscle activation in weak muscles positioned from the origin to the muscle insertion, 15–25% for muscle inhibition caused by overuse or muscle overstretching placed from the insertion towards the muscle origin, and 0–15% for reduction of edema [12]. The best tension of KT to improve proprioception is not evident yet. In the current study, we used the star shape of KT with 15–25% tension because it was reported that the star shape of KT with 15–25% tension significantly improved pain, disability, trunk muscle endurance, and trunk flexion range of motion in patients with nonspecific CLBP [4]. A different method of KT with different tension may enhance lumbar proprioception in patients with CLBP.

Besides, the moderate positive correlation between CE of 15° extension and disability in these patients indicates that those with higher disability display a greater RE. Previous research suggests that spinal RE is the largest among individuals with higher disabilities. Also, there is a positive correlation between functional disability and RE [6, 9]. Those with a higher functional disability may have greater overall disruption of the pain neuromatrix within the CNS [6]. The reasons for nonsignificant correlations between functional disability, pain, and both CE and AE at other angles are unclear. Motor control impairments in spinal posture and movement, as well as trunk muscle activation, have been reported among patients with nonspecific CLBP [6, 43]. Thus, these changes may explain significant or nonsignificant correlations between functional disability, pain, and both CE and AE in the three measured angles, since RE is influenced by muscle spindle feedback [7] and spinal posture [6].

### Limitations

In the current study, some limitations should be considered while interpreting the findings. First, we compared the effects of KT with a placebo group, but the placebo taping was not a real placebo due to the volume of tape used, which should be thinner than KT with less stimulation of subcutaneous afferent sensory fibers. Also, the lack of a non-taping group is another limitation of the current study. Moreover, we only examined the short-term effects of star shape taping with 15–25% tension; however, higher tensions may have different effects on lumbar RE. Therefore, future studies should compare different methods of taping with non-taping, also assess longer follow-ups in patients with CLBP.

### Clinical implication

The use of KT would be beneficial to decrease the level of pain and disability in individuals with nonspecific CLBP. Also, taping without tension even with one strip, such as placebo taping, may reduce pain in these patients. Since the placebo treatments are important tools that can be used by the medical community to complement regular therapies, the use of placebo taping can be helpful. Considering lumbar RE may assist therapists in identifying poor posture awareness and proprioception impairments among these patients.

## Conclusion

The findings suggest that star-shaped KT reduces pain and disability scores after 3 days of application with large effect size in patients with nonspecific CLBP. It also seems that KT may affect another aspect of pain (i.e., sensory, affective) compared to placebo taping, while placebo taping may only improve PPI in these patients. The short-term effect of KT over the low back region cannot improve lumbar proprioception when measured by active lumbar RE. Therefore, the findings do not support the hypothesis that lumbar taping would improve lumbar repositioning errors.

## Data Availability

The datasets generated and analyzed during the current study are available from the corresponding author upon request.

## Abbreviations

CLBP: Chronic Low Back Pain
SD: Standard Deviations
KT: Kinesio tape
CNS: Central Nervous System
RE: Repositioning Error
EMG: Electromyographic
SF-MPQ: Short-Form McGill Pain Questionnaire
S/A: Sensory and Affective
ODI: Oswestry Disability Questionnaire
PPI: Present Pain Intensity
VAS: Visual Analog Scale
ROM: Range of Motion
CE: Constant Error
AE: Absolute Error
NRS: Numerical Rating Scale
MDC: Minimal Detectable Change
